# Roles and mechanisms of ankyrin-G in neuropsychiatric disorders

**DOI:** 10.1038/s12276-022-00798-w

**Published:** 2022-07-06

**Authors:** Sehyoun Yoon, Nicolas H. Piguel, Peter Penzes

**Affiliations:** 1 Department of Neuroscience, Northwestern University, Chicago, IL 60611 USA; 2 Department of Psychiatry and Behavioral Sciences, Northwestern University, Chicago, IL 60611 USA; 3 Northwestern University, Center for Autism and Neurodevelopment, Chicago, IL 60611 USA

**Keywords:** Molecular neuroscience, Phosphorylation

## Abstract

Ankyrin proteins act as molecular scaffolds and play an essential role in regulating cellular functions. Recent evidence has implicated the *ANK3* gene, encoding ankyrin-G, in bipolar disorder (BD), schizophrenia (SZ), and autism spectrum disorder (ASD). Within neurons, ankyrin-G plays an important role in localizing proteins to the axon initial segment and nodes of Ranvier or to the dendritic shaft and spines. In this review, we describe the expression patterns of ankyrin-G isoforms, which vary according to the stage of brain development, and consider their functional differences. Furthermore, we discuss how posttranslational modifications of ankyrin-G affect its protein expression, interactions, and subcellular localization. Understanding these mechanisms leads us to elucidate potential pathways of pathogenesis in neurodevelopmental and psychiatric disorders, including BD, SZ, and ASD, which are caused by rare pathogenic mutations or changes in the expression levels of ankyrin-G in the brain.

## Neuropsychiatry: Potential roles of a scaffolding protein

Mutations affecting the production, distribution, or function of the ankyrin-G protein may contribute to a variety of different neuropsychiatric disorders. Ankyrin-G is typically observed at the synapses between neurons, and contributes to intercellular adhesion and signaling along with other important functions. Peter Penzes and colleagues at Northwestern University, Chicago, USA, review the biology of this protein and identify potential mechanisms by which ankyrin-G mutations might impair healthy brain development. Mutations in the gene encoding this protein are strongly linked with bipolar disorder, but have also been tentatively connected to autism spectrum disorders and schizophrenia. The authors highlight physiologically important interactions with a diverse array of other brain proteins, which can in turn be modulated by various chemical modifications to ankyrin-G, and conclude that drugs that influence these modifications could have potential therapeutic value.

## Introduction

Ankyrin-repeat (ANK) proteins perform various biological functions and are phylogenetically conserved. They are involved in many cellular processes, such as intercellular connections, signal transduction, cell cycle regulation, vesicle transport, inflammatory responses, cytoskeletal integrity, and transcriptional regulation^1^. In humans, 270 ANK proteins have been reported and are significantly correlated with psychiatric risk factors such as BD, ASD, and SZ^2^. An enrichment of ankyrin-repeat domain (ANKRD)-containing protein-encoding genes in BD genome-wide association studies (GWAS) and de novo variants related to ASDs and SZ indicates their broader role in neuropsychiatric diseases^3–7^.

**Fig. 1:**
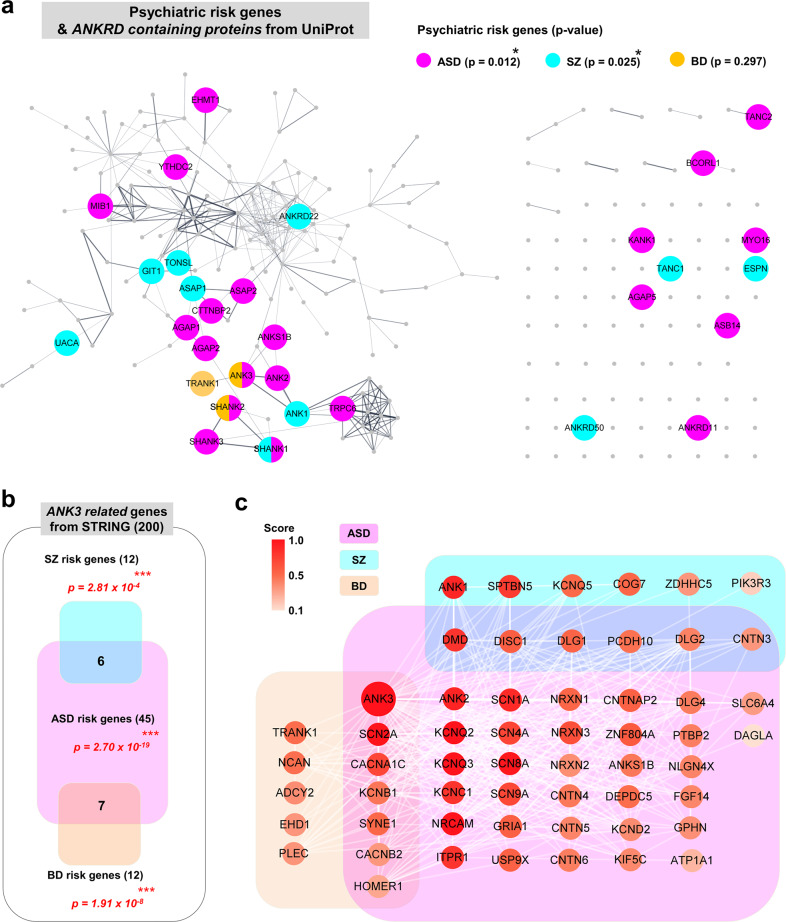
Psychiatric risk factors include ankyrin proteins. **a** Cytoscape analysis of the protein–protein interaction (PPI) network of psychiatric risk genes from GWAS and SFARI and ANKRD-containing proteins retrieved from UniProt and STRING. The large circular nodes indicate psychiatric risk genes, and edge thickness represents the connection score of the connected proteins (0.400 < score < 1.000). *p < 0.05; hypergeometry test (edited from Yoon et al.^2^). **b** Enrichment of SZ risk factors (*n* = 395) (top), BD risk factors (*n* = 155) (bottom) identified through GWAS and ASD risk factors (*n* = 1003) (center) from SFARI among the PPI network of ankyrin-G from STRING (*n* = 200). ***p < 0.001; hypergeometry test. **c** The representative majority of a subnetwork is visualized and annotated by Cytoscape. The color-coded nodes indicate the score factor (0.100 < score < 1.000). SFARI Simons Foundation Autism Research Initiative; STRING Search Tool for the Retrieval of Interacting Genes/Proteins.

We reanalyzed the previously reported correlation between ANKRD-containing proteins and psychiatric risk genes^2^ after adding several recently reported BD risk genes^7^ and 1003 of the ASD risk genes described by the Simons Foundation Autism Research Initiative (SFARI). Significant enrichment of ANKRD-containing proteins was corroborated in ASD and SZ (Fig. 1a). The ANKRD family includes prominent synaptic proteins such as ankyrin-R, -G, and -B (encoded by *ANK1/2/3*), as well as SHANK1/2/3 and TRANK1, which are associated with BDs, ASDs, and SZ. Moreover, ankyrin-G and SHANK1/2 were confirmed to show comorbidities with ASDs in BDs or SZ. Human *ANK3* is related to several neuropsychiatric disorders, including BD, ASD, intellectual disability (ID), and attention-deficit/hyperactivity disorder^8–11^. According to the protein–protein interaction (PPI) network of ankyrin-G from the STRING database, 56 out of 200 related genes (12 genes in SZ, 12 genes in BD, 45 genes in ASD) were identified as significantly included in each psychiatric disorder. Among these, 13 factors were confirmed to exhibit comorbidity (Fig. 1b, c).

In this review, we explore the genetics, spatiotemporal expression patterns, PPI networks, and pathophysiology related to ankyrin-G, which has been highlighted as a high-confidence psychiatric risk gene in extensive genomic studies. Furthermore, we will discuss the possibility of repurposing existing drugs and using current knowledge of the posttranslational modifications of ankyrin-G to develop new drug candidates to treat psychiatric disorders.

## Genetics of *ANK3* and neuropsychiatric disorders

### Bipolar disorder

In 2008, two independent GWASs highlighted single nucleotide polymorphisms (SNPs) in *ANK3*, including rs10994336, rs1938526, and rs9804190, as risk factors for BD^8,12^. A weak linkage disequilibrium between the SNPs was identified, indicating possibly independent contributions of rs10994336 and rs9804190 to the risk of BD^9^. The association between *ANK3* and BD has since been replicated in multiple follow-up GWASs, identifying a range of additional SNPs to further confirm the link between ankyrin-G and psychiatric disease (Supplementary Table 1). The most recent large-scale study analyzed 41,917 individuals with BD and 371,549 controls and again confirmed *ANK3* as a leading BD risk locus (odds ratio = 1.125, *p* = 1.1 × 10^−11^). This study found a large range of SNPs spanning *ANK3* to be associated with BD, all in linkage disequilibrium with rs10994415, the most significant SNP^7^. In addition to the strong genetic links, ankyrin-G polymorphisms have been linked to certain cognitive and anatomic BD subphenotypes; allelic variation in *ANK3* was associated with deficits in sustained attention^13,14^, decision-making^15^, working memory^16^, and verbal comprehension and logical memory^17^. Moreover, magnetic resonance imaging (MRI) studies showed associations of rs10994336, rs9804190, and rs10761482 with the hyperactivation or hypoactivation of specific brain areas^16,18^ and atrophy of white and gray matter in defined brain structures^15,19,20^.

Despite the strong genetic links between *ANK3* and BD, the mechanisms by which risk SNPs affect the expression/function of ankyrin-G and cause disease are not well understood. However, studies have shown that risk SNPs in *ANK3* are correlated with a change in *ANK3* mRNA levels in the blood^21^ and decreased expression of a specific mRNA isoform containing exon 1B in the cerebellum^22^ and that the rs10994336 SNP is associated with a lower methylation level at the CpG site cg02172182^23^. Moreover, a minor isoform of *ANK3* containing exon ENSE00001786716 is increased in BD patients^24^, whereas the loss-of-function mutation rs41283526, which disables correct splicing, has a protective effect^25^. Interestingly, miR34a and miR10b-5p, two microRNAs targeting *ANK3* mRNA, were overexpressed in the postmortem brain^26^ and iPSCs^27^ in BD patients. Altogether, these data suggest a critical role of *ANK3* isoform expression as a risk factor in BD.

Rare variants in the *ANK3* gene, including missense mutations in the promotor or the 3’ untranslated region, have been identified in BD (Supplementary Table 1) from whole-genome or whole-exome sequencing studies^28,29^. Intriguingly, the effect of the Trp1989Arg mutation, found in a family of BD patients, involves disruption of the ankyrin-G and GABARAP interaction, resulting in a decrease in GABAergic synapses^30^.

### Schizophrenia

A GWAS from 2010 found that SNP rs10761482 in the *ANK3* gene was nominally associated with SZ. However, it did not surpass the threshold for genome-wide significance^19,31,32^. Because SZ and BD share risk alleles^33^, several studies have investigated SNP associations from previous BD GWASs in SZ patient cohorts^34–37^ (Supplementary Table 1). Multiple studies have reported that SNPs in the *ANK3* locus are linked to SZ endophenotypes, such as deficits in working memory^38,39^, proneness to anhedonia^40^, and cognitive impairment associated with a reduction in gray matter^41^. The most recent large-scale GWAS for SZ did not show a significant association between *ANK3* and SZ^42^. However, a recent GWAS from patients with psychotic experiences, including those with BD and SZ, found an association for two *ANK3* loci, an intronic variant (rs10994278) and an intergenic variant (rs549656827)^43^. Thus, the link between SZ and *ANK3* remains unclear, and further work is required to determine whether a link between *ANK3* and SZ or SZ subtypes exists.

### Autism spectrum disorders

In contrast with BD and SZ, most genetic studies on ASD have focused on rare de novo mutations rather than GWAS; therefore, no common variants have yet been found linking *ANK3* with ASD, but a multitude of rare de novo mutations in ASD probands have been reported (Supplementary Table 1). Missense mutations were found in exons 5, 6, 30, and 37 of *ANK3*, and several frameshift mutations were also identified^4,10,11,44–51^. In addition, rare mutations in *ANK3*, *CREBBP*, and *SEMA6B* were identified in an ASD patient, inherited from both parents. Each mutation was associated with increased gene expression, and the mutations could act synergistically to modulate disease severity^45^.

## Multiple isoforms of ankyrin-G in the brain

The *ANK3* gene is localized to human chromosome 10q21 between 61.4 and 62.2 million base pairs. Five alternative first exons have been identified for human *ANK3*, namely, exons 1a, 1b, 1e, 1f, and 1s^22^. Isoforms including exon 1e and exon 1b were found to be expressed at similar levels in the frontal cortex and cingulate cortex and accounted for almost all *ANK3* expression. However, *ANK3* exon 1b was expressed at approximately 3−4-fold higher levels in the cerebellum than in the frontal or cingulate cortex^22^. In C57BL/6J mice, exon 1b expression was also highest in the cerebellum and was substantial in the dentate gyrus and all other regions examined (hippocampus, amygdala, orbitofrontal cortex, PFC, and striatum)^52^. *Ank3* exon 1b transcripts were expressed at higher levels in all brain regions than the alternative leading exon 1e, expressed in transcripts found in the brain and other tissues^53^. Moreover, exon 1b expression was limited to oligodendrocytes, whereas exon 1b- and 1e-containing ankyrin-Gs were expressed in neurons^54^.

**Fig. 2:**
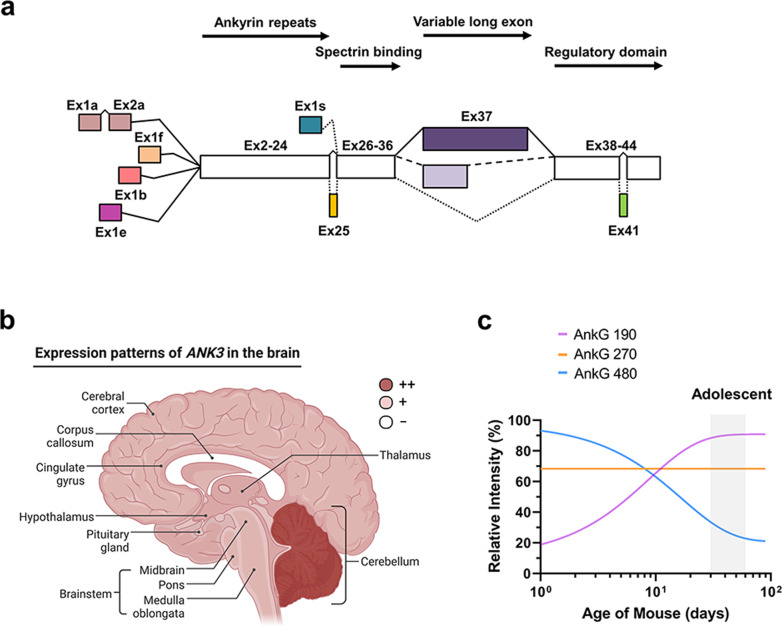
Spatiotemporal expression of different isoforms of *ANK3* in the brain. **a** A diagram of *ANK3* transcription start sites and alternative splicing. **b** Expression patterns of the *ANK3* gene in the human brain (adapted from The Human Protein Atlas). **c** A schematic summary of the trajectories of the different ankyrin-G isoforms in the mouse brain. Expression levels were measured as log_10_ of western blot intensities from Yoshimura et al.^61^.

There are five isoforms for human *ANK3* in the RefSeq database encoding ankyrin-G. Three of these, one weighing 202 kDa and two weighing 204 kDa, have a domain structure that includes ANK repeat, spectrin-binding, and regulatory domains. These three protein isoforms are collectively referred to as ankyrin-G 190 because they migrate at 190 kDa by sodium dodecyl sulfate–polyacrylamide gel electrophoresis (SDS–PAGE). Ankyrin-G 190 localizes to postsynaptic sites and dendritic spines, modulating dendritic spine morphology and synaptic transmission at glutamatergic synapses^55^. The fourth form has an additional large exon 37 (Ensembl ID ENSE00000997921), which encodes a serine-rich domain. This large exon can be spliced in 2 distinct ways, and the resulting transcripts are referred to as the ankyrin-G 270/480 isoforms (Fig. 2a). The isoform with the shorter *ANK3* exon 37 (ankyrin-G 270) is preferentially expressed in the frontal and cingulate cortex, whereas the isoform with the longer *ANK3* exon 37 (ankyrin-G 480) has higher expression in the cerebellum^22^. Alternative splicing modifications of exon 37 are detected only in brain tissue, and these different isoforms are known to have diverse functions at different cellular locations. The fifth isoform has the lowest molecular weight, 110 kDa, and completely lacks the ankyrin-repeat domain. The expression of this isoform, starting from exon 1 s, within specific regions in the brain is poorly understood. Functionally, it has been previously reported that this isoform regulates endocytosis and lysosomal degradation^56^. Of note, exon 41 (Ensembl ID ENSE00001765562) has been identified as an exon inserted into the 190 and 110 kDa isoforms of ankyrin-G, and the Homer1b/c or p85 subunit of PI3K proteins are known to bind to this inserted site^56,57^.

## Expression patterns of ankyrin-G in the brain

*ANK3* is most highly expressed in the cerebellum but is generally expressed in other brain regions, such as the cerebral cortex, hippocampus, corpus callosum, and hypothalamus (Fig. 2b; from The Human Protein Atlas project). These results are consistent with previous reports analyzing the expression pattern of *ANK3* mRNA in the brains of mice^52,53^. In 3-month-old wild-type mice, ankyrin-G is expressed in 40% of cells in the somatosensory cortex from layer II to V and in 57% of cells in the CA3 of the hippocampus^2^. Analyses of conditional *Ank3* knockout mice (AnkG cKO) with homozygous loss of ankyrin-G in forebrain pyramidal neurons, 70% in the somatosensory cortex and 95% in the CA3 of the hippocampus, indicate that ankyrin-G-expressing cells could be excitatory neurons^58,59^. These decreases were also observed in the piriform and cingulate cortices. A certain percentage of cells positive for ankyrin-G coexpressed with parvalbumin was observed in the AnkG cKO brain, confirming that ankyrin-G is also expressed in inhibitory neurons^58^. Considering the critical role of synaptic dysfunction in neuropsychiatric disorders, we suggest that at least some phenotypes arise from ankyrin-G-mediated disorders, alterations in the excitatory−inhibitory (E/I) balance, and circuit disturbances in forebrain regions. In addition, specific ankyrin-G in oligodendrocytes is essential for the enrichment of myelin sheaths surrounding the nodes of Ranvier (NoR)^54^.

## Developmental expression patterns of ankyrin-G isoforms

The developmental transcriptome dataset from BrainSpan illuminates how the expression of exons changes from infancy to adulthood. Human exon 25 (Ensembl ID ENSE00001786716) from the RNA-seq dataset has a low level of expression during infancy. However, its expression increases at the age of 11 and reaches a plateau throughout adolescence and adulthood. The expression of exon 24 (Ensembl ID ENSE00000997958) is also low, similar to that of exon 25, in the fetal period. However, its expression gradually increases from childhood to reach a considerably higher level throughout adolescence and adulthood^25^.

Immunohistochemical observation of the PFC of a rhesus monkey showed that ankyrin-G expression in the axon initial segment (AIS) decreased from birth until 3 months of age. The observation then shows a pattern of stabilizing expression from adolescence to adulthood^60^. These results were consistent with the results of a western blot observations of mouse whole-brain lysate. Ankyrin-G 480, expressed in the AIS, continuously decreases after birth and stabilizes through adolescence, whereas ankyrin-G 270 is evenly expressed throughout life^61^. Interestingly, in the case of ankyrin-G 190, the expression continuously increases after birth, and the expression level is maintained at a constant level after adolescence and into adulthood (Fig. 2c). These results suggest that three major isoforms in the brain, ankyrin-G 190 and ankyrin-270/480, play their respective roles at distinct locations in neurons throughout the developmental period.

## Structure of ankyrin-G and its binding partners

### ANKRD

Ankyrin-G, as a scaffolding protein, plays a pivotal role in regulating the localization of ion channels, membrane transporters, cell adhesion molecules, and cytoskeletal proteins. Here, we introduce the ANKRD-binding partners of ankyrin-G.

#### Ion channels/transporters/pumps

It has been reported that ankyrin-G plays an important role in response to physiological signals by interacting with various types of membrane transporters through ANKRD. Cyclic nucleotide-gated (CNG) channels, such as the rod photoreceptor cGMP-gated cation channel, can localize to the plasma membrane by binding to ankyrin-G through the cytoplasmic C-terminal domain of the channel β1 subunit^62^. Ankyrin-G is exclusively localized to rod outer segments (ROSs) and is required for the transport of CNG-β1 and ROS morphogenesis. Ankyrin-G also modulates the localization of other membrane transporters, such as sodium/potassium-transporting ATPase subunit alpha-1 (Na +/K + ATPase α1), anion exchanger 1 (AE1), and ammonium transporter Rh type B (RhBG), via interaction with their cytosolic domains^63–66^. Exons 13–24 of ANKRD are interaction sites for AE1 and RhBG^66^. Binding to ankyrin-G is necessary for voltage-gated sodium channels to form clusters at the AIS and the NoR. Ankyrin-G uses its ANKRD to bind to members of the Nav1 family by recognizing a 9-amino-acid motif ((V/A)P(I/L)AXXE(S/D)D) in the cytosolic loop that links transmembrane domains II and III in these channels^67,68^. Ankyrin-G additionally interacts with potassium channels Kv7.2 (KCNQ2) and Kv7.3 (KCNQ3) via a conserved stretch of 10 amino acids, which is similar to the ankyrin-G-binding sequence in Nav1 channels^69^.

#### Cell adhesion molecules

Ankyrin-G is known to regulate the organization of the AIS and NoR through interaction with cell adhesion molecules such as CHL1, NRCAM, and neurofascin^70^. The conserved FIGQY motif located in the C-terminal cytosolic part of L1CAM family members is essential for binding to ankyrin-G^71^. Another type of cell adhesion molecule, E-cadherin, requires both ankyrin-G and β-2-spectrin for its cellular localization in early embryos and binds to ankyrin-G. Ankyrin-G interacts with the cytoplasmic tail (amino acids 738–764) of E-cadherin and provides a direct connection between E-cadherin and the spectrin/actin skeleton^72^. N-cadherin also expresses this highly conserved motif, and ankyrin-G binds N-cadherin and E-cadherin equally. Moreover, ankyrin-G is localized at neuromuscular junctions and costameres within skeletal muscle and binds β-dystroglycan (β-DG) and dystrophin^73^. Ankyrin-G binds β-DG as well as dystrophin and is required to restrict β-DG and dystrophin to costameres^74^. Ankyrin-G is also required for sarcolemmal integrity to organize β-DG and sarcolemmal dystrophin. The adhesion molecule CD44 interacts with 7–12 ANKRD (repeats 7–12) and binds to hyaluronic acid at its extracellular domain. This interaction can regulate prostate tumor cell transformation^75^ or ovarian tumor cell migration^76^.

#### Scaffold proteins/motor proteins/enzymes

The IQ motif containing J-Schwannomin-Interacting Protein 1 (IQCJ-SCHIP-1), an isoform of SCHIP-1, is highly enriched at the AIS, and its accumulation is dependent on the ANKRD of ankyrin-G^77^. IQCJ-SCHIP1 is associated with molecular complexes consisting of voltage-gated Nav, Kv7 channels, and cell adhesion molecules, including βIV-spectrin. IQCJ-SCHIP-1 self-associates through its C-terminus, and this association orchestrates multimolecular complexes composed of Nav/Kv7 channels, cell adhesion molecules, βIV-spectrin, and ankyrin-G in the AIS^78^.

Ankyrin-G associates with the anterograde motor KIF5 and Nav1.2 channel together. KIF5 binds to ankyrin-G by interacting with ANKRD 1–6 and amino acids 865–934 in the C-terminus of KIF5^79^. Nav1.2 in the AIS was markedly reduced by the knockdown or knockout of ankyrin-G as well as the disruption of KIF5 binding to ankyrin-G through the overexpression of dominant-negative KIF5 constructs. The ankyrin-G-KIF5 complex is essential for the anterograde transport of Nav1.2 in the AIS and modulates action potential firing.

T-lymphoma invasion and metastasis 1 (Tiam1) is a guanine nucleotide exchange factor for Rho GTPases and interacts with ANKRD through amino acids 717–727 of Tiam1 (GEGTDAVKRS). Ankyrin-G binding to Tiam1 regulates the membrane localization of Tiam1 and activates a GDP/GTP exchange on Rho GTPases to regulate cytoskeletal function during cell migration^80^.

Recently, we found that Ubiquitin-Specific Peptidase 9 X-Linked (USP9X) modulates spine morphology through ankyrin-G stabilization^2^. Pathogenic USP9X variants were discovered in patients diagnosed with autistic and obsessive behaviors, delayed speech, developmental delay (DD), brain malformations, and growth retardation^81–83^. In particular, human G1890E mutation from 2 patients with ID, DD, speech delay, and ASD were found to have normal catalytic activity but impaired interaction with ankyrin-G^2^. The deubiquitination of proteins containing ANKRD is important for the accurate development of synapses, as a lack of USP9X results in the malfunction of synaptic structural plasticity as well as behavioral and clinical abnormalities. Notably, ankyrin-B, Shank3, and TNKS2 levels also significantly decreased in USP9X^–/Y^ mice; male offspring that inherited the Emx1-Cre allele lacked USP9X in the telencephalon and derived cortex and hippocampal structures, indicating that USP9X stabilizes multiple ANKRD proteins in vivo.

We also reported that diacylglycerol lipase α (DAGLα) (encoded by *DAGLA*), which synthesizes the endocannabinoid 2-arachidonoyl-glycerol (2-AG), interacts with the ANKRD of ankyrin-G through C-terminal amino acids 709–847 (detected in a yeast-2-hybrid [Y2H] experiment). The DAGLα-ankyrin-G interaction regulates spine morphology and the lateral diffusion of DAGLα in neuronal dendrites^59^. Remarkably, 3 rare heterozygous variants in human *DAGLA* (His810Gln, Arg815His, and Ala858Val) have been identified in patients presenting with seizures and neurodevelopmental disorders, including ASD, as well as abnormalities in brain morphology^84^. 2-AG is a mediator of retrograde signaling to presynaptic CB1 receptors to regulate the release of neurotransmitters. However, we found that the C-terminal region of DAGLα mediated a putative nonretrograde pathway that regulates dendritic spine development via interaction with ankyrin-G.

### Spectrin-binding domain

The 2 ZU5 domains and a UPA domain (ZU5^N^-ZU5^C^-UPA), followed by ANKRD, interact with spectrin/actin^85^. This motif has been highly conserved in ankyrin-G and across species. The DAR999AAA or Ser2417Ala mutations in ankyrin-G 480 abolished ankyrin-G binding to βII-spectrin^86,87^. βIV-spectrin is also known to be a component of dendritic spines. Interestingly, the DAR999AAA mutation in ankyrin-G 190 prevented its localization to the spine head, which reduced spine development and maintenance^55^.

### Giant exon inserted domain

The neuron-specific exon 37 of ankyrin-G is inserted after the spectrin-binding domain. The resulting giant ankyrin-G transcripts are specifically expressed in the brain, and they encode ankyrin-G isoforms that migrate at 270 and 480 kDa by SDS–PAGE (ankyrin-G 270/480). The ankyrin-G 270/480 isoforms are highly enriched in AIS and are critical for organizing multiple AIS-associated proteins, including cell adhesion molecules, β-spectrins, and ion channels^88^. Ankyrin-G 480 interacts with GABARAP or GABARAPL1 through the Atg8 interaction motif. It regulates the stabilization of gamma-aminobutyric acid type A (GABAA) receptors at somatodendritic sites and the formation of GABAergic inhibitory synapses^87,89,90^. Furthermore, ankyrin-G 480 directly associates with end-binding proteins via its specific tail domain (exon 37); this interaction is crucial for AIS formation and neuronal polarity^91,92^. Moreover, the dynein regulator NDEL1 controls somatodendritic cargo transport at the AIS via an interaction with the specific tail domain of ankyrin-G^93^.

### Regulatory domain

The C-terminal regulatory domain is composed of a highly conserved death domain (amino acids 1478–1562) and an unstructured stretch of 400 amino acids. Using a Y2H screen as a bait for the death domain of ankyrin-G 190, the proapoptotic molecules Fas and FADD were identified in kidney epithelial cells and confirmed by immunoprecipitation, colocalization, and pull-down assays^94^. This suggests that ankyrin-G may contribute to apoptotic cell death.

The EVH1 domain of Homer1b/c recognizes the PPXXF motif of ankyrin-G 190, a protein that is highly enriched in the PSD and is responsible for regulating spine morphology and maintaining a stable ankyrin-G pool in the spine head^57^. The ankyrin-G interaction partner DAGLα is also known to interact with Homer1 through the PPXXF motif in its C-terminal region^95^. These data suggest that Homer1 may regulate synaptic localization and function in complex with its binding partners within spine heads. A smaller isoform (110 kDa) of ankyrin-G lacks the membrane domain and binds directly to the p85 subunit of PI3K through both the spectrin domain and the regulatory domain of ankyrin-G. This isoform is localized to late endosomes and lysosomes and regulates signaling pathways downstream from platelet-derived growth factor receptor (PDGFR) by lysosomal-mediated degradation of tyrosine-phosphorylated PDGFR through an interaction with the p85 subunit of PI3K^56^.

### Interactors with an unknown binding domain

Various additional interacting partners, such as GluA1, dystrophin, Hook1, and Plakophilin-2, have been found by immunoprecipitation experiments^55,74,96,97^, although their exact binding sites within ankyrin-G have not yet been identified.

## Posttranslational modifications in ANKRD and its functional effects on ankyrin-G and binding partners

### Palmitoylation

Palmitoylation is a reversible posttranscriptional modification consisting of a saturated fatty acid chain on cysteine residues through thioester bonds. This modification impacts protein hydrophobicity and allows the target protein to interact with the lipid bilayer. Genes within the zDHHC family, which includes 23 genes in humans, encode protein S-acyltransferases (PATs) that have specific interactions with their substrates and different distributions in cell compartment membranes^98,99^. In the brain, zDHHC proteins play various roles, including in neurite outgrowth and in synaptic plasticity, by targeting the membrane and conferring different subcellular localizations, such as to dendritic spines. Importantly, zDHHC proteins are associated with various brain disorders^100,101^.

**Fig. 3:**
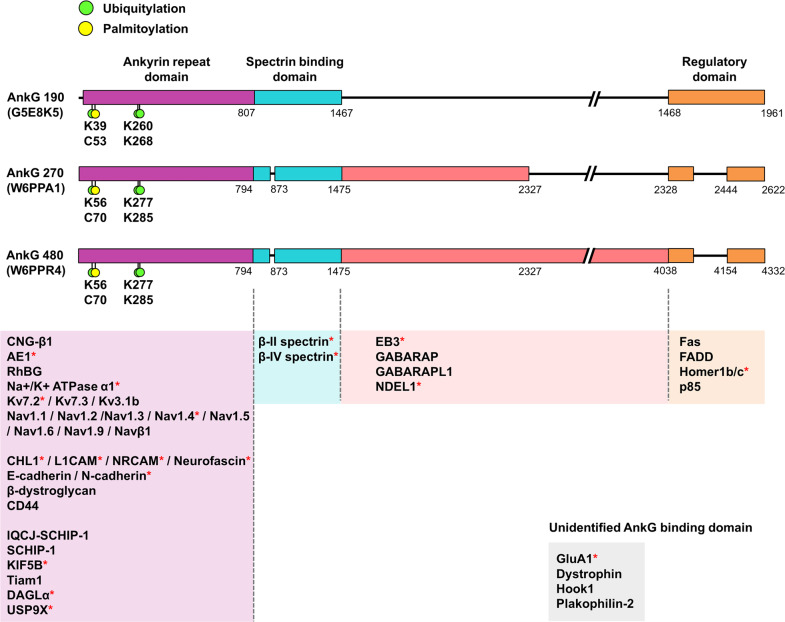
Posttranslational regulation of ankyrin-G and structure-dependent ankyrin-G binding partners. Ankyrin-G forms four distinct domains: an ankyrin-repeat domain (ANKRD), a spectrin-binding domain, a regulatory domain that includes a death domain (DD), and a C-terminal domain (CTD). Each domain interacts with distinct ion channels, transporters, cell adhesion proteins and signaling molecules, and cytoskeletal elements. *: PSD proteins.

To accomplish their roles as scaffold proteins, the different ankyrin-G isoforms rely on their localization in membrane microdomains^102^. Although several cysteines in ankyrin-G have been predicted as potential palmitoylation sites (SwissPalm), only cysteine 70 has been functionally validated^103^ (Fig. 3). X-ray crystallography and molecular dynamics simulations show that ankyrin-G has a preferential localization close to the membrane and that its palmitoylation stabilizes its localization to the membrane^104^. In epithelial cells, the absence of ankyrin-G palmitoylation prevents membrane localization under low-calcium conditions and abolishes ankyrin-G function in lateral membrane biosynthesis. In neurons, cys70 palmitoylation is important for the proper localization of ankyrin-G 270 in the AIS and the recruitment of AIS components^103^. This also stabilizes ankyrin-G 190 in the microdomains of dendritic spines and along the dendritic membrane^105^.

A screen performed in HEK293 cells with the 23 PATs showed that zDHHC5 and zDHHC8 could palmitoylate ankyrin-G and were essential for its localization at the membrane of Madin-Darby canine kidney cells^106^. In neurons, only zDHHC8 was involved in ankyrin-G 190 localization in dendritic spines^105^, and further studies are needed to precisely define the effects of those PATs on ankyrin-G isoforms and functions. Although zDHHC5 plays an essential role in excitatory synapses^107^, notably through GRIP1^108^, few studies have linked it to SZ through GWAS^109^ or de novo variants^3^. In contrast, zDHHC8 has been identified as an SZ susceptibility gene, especially in 22q11.2 deletion syndrome^110^. Further studies are necessary to comprehensively understand the link between PATs and the ankyrin-G pathway in disease.

### Ubiquitination

Twenty of the ANKRD proteins, which are also neuropsychiatric factors, have ubiquitinated lysine in ANKRD^2^. These proteins have highly conserved D boxes, which are the recognition motifs of E3 ligases. Deubiquitylating enzymes (DUBs) play key roles in stabilizing substrates by removing mono- or polyubiquitination. A recent report showed that USP9X regulates the homeostasis of ankyrin-G in early cortical neuron development^2^ and that this lack of USP9X causes abnormal brain morphology^111^. In fact, in 44 USP9X patients, 12 missense variants on the X-chromosome were identified, and these patients were reported to have speech delays and various behavioral problems^83^. These results indicate that the ubiquitylation signaling pathway (USP) and DUB systems coordinate various neurodevelopmental events by regulating the homeostasis of ANKRD proteins in the brain and that genetic variants can cause neurodevelopmental disorders along with various neuropsychiatric diseases.

### Phosphorylation

**Fig. 4:**
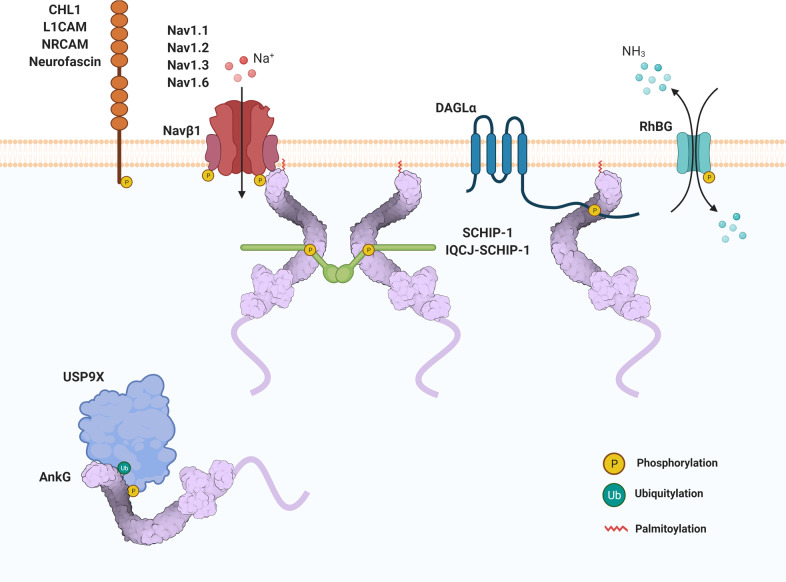
A schematic model of the interacting partners of ankyrin-G, drawn with BioRender. The model shows the interacting partners of ankyrin-G regulated by phosphorylation.

Phosphorylation is well known to play a critical role in PPI, including in ANKRD proteins; in particular, specific phosphorylation motifs have been reported in proteins interacting with ankyrin-G (Table 1 and Fig. 4).

**Table 1:** Phosphorylation motifs of proteins interacting with ankyrin-G.

Gene symbols	Protein ID	Protein function	Interaction motif	Phosphorylation sites	Regulatory signaling molecules	Affinity	Related-psychiatric disorder
*CHL1*	O00533	Cell adhesion	1174–1185	Y1185	−	↓	−
*L1CAM*	P32004	Cell adhesion	1218–1229	Y1229	EphB1/2/3	↓	−
*NFASC*	O94856	Cell adhesion	1308–1319	Y1319	FGF2, NGF	↓	−
*NRCAM*	Q92823	Cell adhesion	1265–1276	Y1276	−	↓	ASD
*RHBG*	NP_065140.3	Membrane transporter	419–421	Y429	Src and Syk kinases	↓	−
*SCN1B*	Q07699	Ion channel	183–200	Y200	FGF2, FYN	↓	−
*SCN1A*	P35498	Ion channel	1112–1138	S1122; S1134; S1136	CK2A1	↑	ASD
*SCN2A*	Q99250	Ion channel	1102–1128	S1112; S1124; S1126	CK2A1	↑	ASD, BD
*SCN3A*	Q9NY46	Ion channel	1100–1126	S1110; S1122; S1124	CK2A1	↑	−
*SCN8A*	Q9UQD0	Ion channel	1093–1119	S1103; S1115; S1117	CK2A1	↑	ASD
*SCHIP-1*	P0DPB3	Protein binding	307–352	N/I	CK2	↑	−
*IQCJ-SCHIP-1*	B3KU38	Protein binding	383–428	N/I	CK2	↑	−
*DAGLA*	Q9Y4D2	Enzymatic activity	709–847	S737	PKA	↑	ASD
*USP9X*	Q93008	Enzymatic activity	1719–1842	S1593; S1609	TGFβ, Insulin	↑	ASD

*N/I* not identified.

The ANKRD of ankyrin-G binds to cell adhesion family members (NRCAM, CHL1, Neurofascin, L1CAM) through the conserved amino acid sequence FIGQY in the cytosolic domain. The phosphorylation of the tyrosine in the FIGQY of cell adhesion proteins prevents its interaction with ankyrin-G^71,112,113^. EphrinB1 phosphorylates the tyrosine of cell adhesion family proteins at FIGQY via EphB and Src kinase activity, and the Src kinase inhibitor PP2 decreases the phosphorylation of L1CAM^114^. The tyrosine phosphorylation induced by ephrinB1 reduces the interaction with ankyrin-G, enhancing axonal growth. In contrast, the dephosphorylation of FIGQY through tyrosine phosphatases promotes ankyrin-G interactions and stabilizes synaptic terminals through a spectrin/actin cytoskeleton. Neurofascin is also known to regulate phosphorylation by activating tyrosine kinase through fibroblast growth factor-β (FGF2) or nerve growth factor or upon the enhancement of tyrosine phosphorylation through protein tyrosine phosphatase inhibitors such as dephostatin or vanadate^112^.

The C-terminus of RhBG is essential for its localization to the basolateral membrane of kidney epithelial cells by tyrosine phosphorylation, and dephosphorylation allows its interaction with ankyrin-G to connect to the spectrin/actin cytoskeleton^65^. Tyrosine 429 is phosphorylated by Src and Syk kinases. Phosphorylation is enhanced by treatment with pervanadate, a protein tyrosine phosphatase inhibitor. The phosphorylation of RhBG Tyr429 abolishes the interaction with ankyrin-G and decreases its plasma membrane stability.

The phosphorylation of voltage-gated sodium channels by the protein kinase CK2 regulates their interaction with ankyrin-G, resulting in their accumulation at the AIS. Serine sites within the conserved motif (TVTVPIAVGE**S**DFENLNTEDFS**S**E**S**DL) in the second loop of sodium channels (SCN1A, SCN2A, SCN3A, and SCN8A) are phosphorylated by CK2A, and this phosphorylation enhances their interactions with ANKRD^68,115^. Another auxiliary subunit of the sodium channel SCN1B undergoes tyrosine phosphorylation by FGF2 or FYN kinase in the cytosolic C-terminal region, and this phosphorylation ameliorates the interaction with ankyrin-G^116^. The association of ankyrin-G with Nav1 and KCNQ2/3 is altered by protein kinase CK2-mediated phosphorylation.

CK2-mediated phosphorylation of IQCJ-SCHIP-1 enhances ankyrin-G and IQCJ-SCHIP-1 interactions. Several phosphorylation sites in IQCJ-SCHIP-1 or SCHIP-1a were observed by a C-terminal tail truncation mutant of SCHIP-1, and the phosphorylation of these sites directly bound to ankyrin-G. The CK2 inhibitor 4,5,6,7-tetrabromobenzotriazole reduced IQCJ-SCHIP-1 and ankyrin-G accumulation in AIS^117^. The CK2-mediated IQJC-SCHIP-1 association with ankyrin-G contributed to AIS maintenance.

Forskolin treatment increases intracellular cyclic adenosine monophosphate (cAMP) levels, activating protein kinase A (PKA) signaling and initiating a signaling cascade leading to DAGLα serine 738 phosphorylation. In this signaling mechanism, the phosphorylation of serine 738 is inhibited by treatment with RP-8-Br-cAMPS, a PKA inhibitor. Forskolin treatment-induced serine 738 phosphorylation of DAGLα enhances its interaction with ankyrin-G, increasing spine size and decreasing DAGLα surface diffusion^59^. These results suggest that cAMP-mediated acute spine enlargement is regulated through an independent retrograde endogenous cannabinoid system^59^.

USP9X is a key regulator of the transforming growth factor-β (TGFβ) signaling pathway^118^, regulating dendritic development and neuronal axonal growth in response to TGFβ stimulation^119^. TGFβ receptors, or serine/threonine kinase receptors, activate downstream signaling cascades such as the extracellular signal-regulated kinase, phosphoinositide 3-kinase, mitogen-activated protein kinase, Smad family, AKT, nuclear factor-kB, and JUN N-terminal kinase cascades^120^. TGFβ treatment phosphorylates serine 1593 and 1609 in USP9X, and these modifications enhance the interaction with ankyrin-G to modulate spine morphology^2,121^. TGFβ treatment also phosphorylates serine 1600 and regulates USP9X activity rather than binding to ankyrin-G^122^. In addition, insulin is known to phosphorylate serine 1593 and is inhibited by MK-2206 (Akt1/2/3 inhibitor)^123^. This suggests that DUBs may be an ideal drug target for the enhancement or inhibition of substrate binding^124^. Recently, an optogenetic system (optoTGFBRs) was engineered to precisely control TGF-β signaling temporally and spatially^125^. Using this system, it is possible to selectively control signal activity through light stimulation of specific cells or tissues at the desired time. In the future, these techniques may be useful in developing therapeutic approaches to treat human psychiatric disorders.

## Conclusion

ANKRD proteins are structurally capable of performing various intracellular physiological functions by binding to partner proteins. In particular, several isoforms of ankyrin-G in the brain are formed through alternative splicing and play significant roles in different intracellular locations. In addition, these isoforms are expressed quantitatively at different developmental periods to regulate neuronal development. The ankyrin-G-related PPI network is significantly involved in neuropsychiatric diseases such as ASD, BD, and SZ. These results suggest that the modification of ankyrin-G and related proteins is a potential therapeutic approach for neuropsychiatric disorders. In the future, further studies should be performed to increase our understanding of these detailed mechanisms, and they will be helpful in the development of personalized treatments for individual patients with fewer side effects.

## Supplementary information

Supplemental Table 1
