# Supplementary material for: Roles and mechanisms of ankyrin-G in neuropsychiatric disorders
Source: Exp Mol Med. 2022 Jul 6;54(7):867–77. doi: 10.1038/s12276-022-00798-w (PMC9356056; doi:10.1038/s12276-022-00798-w)
Supplement: Supplementary file 1 — Supplemental Table 1 [file 12276_2022_798_MOESM1_ESM.pdf]

Supplementary Table 1. SNPs and mutations in ANK3 loci

GWAS (BD)

SNP name	bp position (hg 38)	RefSeq NM_020987	RefSeq NM_001204403	First Bipolar Disorders GWAS			Mullins et al. 2021	Link with Schizophrenia		
				reference	p value	OR	p value	reference	p value	OR
**rs10994430(G>T)**	chr10:60612195	Genic Upstream	Intron2	Mühleisen et al 2014	2.14E-08	1.18	>10-8	-		
rs10994415 (C>T)[#]	chr10:60562276	Genic Upstream	Intron2	Mühleisen et al 2014	6.97E-10	1.18	1.1E−11 OR=1.125	-		
**rs1938526 (A>G)**	chr10:60540625	Genic Upstream	Intron2	Ferreira et al 2008	1.30E-08	1.395	>10-8	Hong Lim 2014	1.20E-02	0.6
**rs1938540 (T>C)**	chr10:60535056	Genic Upstream	Intron2	Mühleisen et al 2014	8.22E-10	1.27	>10-8	-		
**rs10994397 (C>T)**	chr10:60519366	Genic Upstream	Intron2	Sklar et al 2011	7.08E-09	1.35	7E−9	-		
**rs10994359 (T>C)**	chr10:60462349	Genic Upstream	Intron2	Ripke et al 2011	2.45E-08	1.22	3E−9	Ripke 2011	2.45E-08	1.22
**rs4948418 (C>T)**	chr10:60425736	Genic Upstream	Intron2	Chen et al 2013	8.93E-09	-	4E−10	Guo2016	7.90E-02	1.86
**rs10994338 (G>A)**	chr10:60421370	Genic Upstream	Intron2	Mühleisen et al 2014	3.44E-08	-	>10-8	Guo 2016	8.40E-02	1.84
**rs10994336 (C>T)**	chr10:60420054	Genic Upstream	Intron2	Ferreira et al 2008	9.1E-09	1.45	9E−9	Yuan 2012	1.00E-04	1.4
**rs10821745 (G>T)**	chr10:60376448	Intron1	Intron2	Mühleisen et al 2014	1.26E-08	1.27	>10-8	-		
**rs10994318 (C>G)**	chr10:60366098	Intron1	Intron2	Stahl et al 2019	6.8E10-09	1.145	>10-8	-		
**rs10821736 (T>C)**	chr10:60345295	Intron1	Intron2	Mühleisen et al 2014	1.55E-08	1.28	>10-8	-		
**rs10994415 (C>T)**	chr10:60339194	Intron1	Intron2	Mühleisen et al 2014	6.88E-11	1.27	>10-8	-		
**rs10994308 (A>G)**	chr10:60339194	Intron1	Intron2	Mühleisen et al 2014	3.59E-08	-	>10-8	-		
rs10761482(C>T)	chr10:60325579	Intron1	Intron2	-			-	Athanasiu 2010	7.68E-06	-
**rs10509129 (T>G)**	chr10:60311283	Intron1	Intron2	Mühleisen et al 2014	4.77E-08	1.29	>10-8	-		
rs9804190 (C>T)	chr10:60080073	Intron36	Intron36	Baum et al 2008	2E-03	-	-	Roussos 2012	-	-

bold SNPs r^2>0.6 with rs10994415 in Mullins 2021

[#]SNP most significant in Mullins 2021

Rare variants (BD)

SNP name	bp position (hg 38)	Base change	RefSeq NM_020987.5	RefSeqNM_001204403.2	Inheritance	Publication	Disease
-	chr10:60055693	C>T	exon42:p.His4344Tyr	exon42:p.His1828Tyr	inherited	Forstner et al 2020	BD
rs140741466	chr10:60073412	C>T	exon37:p.Pro2490Leu	Intron36	inherited	Forstner et al 2020	BD
rs41283526	chr10:60145969	T>C	Intron 23	Intron24[#]	-	Hugues et al 2016	BD
rs372922084	chr10: 60074916	T>C	Exon37 p.Trp1989Arg	Intron36	inherited	Nelson et al 2018	BD
rs139972937	chr10:60072953	A>G	Exon37 p.Asp2643Ser	Intron36	inherited	Fiorentino et al 2014	BD
rs370916512	chr10:60028868	C>A	3'UTR	3'UTR	-	Fiorentino et al 2014	BD
rs184389434	chr10:60734079	A>T	Promotor	Promotor	-	Fiorentino et al 2014	BD

[#]exon 24 for NM_001204404

Rare variants (ASD)

SNP name	bp position (hg 38)	Base change	RefSeq NM_020987.5	RefSeqNM_001204403.2	Inheritance	First publication
rs112339619	chr10:60734058	C>T	Genic Upstream	Genic Upstream	de novo	McKenna et al 2018
-	chr10:60615200	C>T	Genic Upstream	exon2:p.Arg28*	inherited	Wang et al 2016
-	chr10:60279627	delG	exon2: p.Ala43Glnfs*4	exon3: p.Ala37Glnfs*4	de novo	Kloth et al 2021
-	chr10:60270201	C>T	exon5:p.Ala148Val	exon6:p.Ala148Val142	de novo	Feliciano et al 2019
-	chr10:60263890	C>T	exon6:p.Thr215Met	exon7:p.Thr209Met	de novo	Chen et al 2017
-	chr10:60261888	delC	exon7: p.Arg257*	exon8 :p.Arg251*	-	Kloth et al 2021
rs267602542	chr10:60205850	G>A	exon11:p.Arg412Gln	exon12:p.Arg406Gln	inherited	Wang et al 2016
-	chr10:60200172	G>A	exon13:p.Arg483Gln	exon14:p.Arg477Gln	inherited	Stressman et al 2017
-	chr10:60196195	G>C	exon16:p.Val613Leu	exon17:p.Val607Leu	inherited	Wang et al 2016
rs1591451043	chr10:60186810	G>T	exon17: p.Gly664*	exon18: p.Gly658*	de novo	Kloth et al 2017
-	chr10:60186747	delG	exon17: p.Leu684Serfs*7	exon18: p.Leu678Serfs*7	-	Kloth et al 2021
rs190581397	chr10:60173110	C>T	exon19:Ala754Val	exon20:p.Ala748Val	inherited	Wang et al 2016
rs191792213	chr10:60172916	C>T	exon20:p.Pro789Leu	exon21:p.P783L	inherited	Stressman et al 2017
rs202156367	chr10:60166859	C>T	exon22:p.Thr839Met	exon23:p.Thr833Met	-	Stressman et al 2017
rs775705277	chr10:60134356	C>T	exon25:p.Ser919Leu	exon26:p.Ser913Leu	-	Stressman et al 2017
-	chr10:60116218	-	intron25: t(2;10)(q11.2;q21.2)	intron26: t(2;10)(q11.2;q21.2)	de novo	Iqbal et al 2013
rs730882195	chr10:60114271	G>C	exon26:p.Asp968His	exon27:p.Asp962His	-	Stressman et al 2017
rs761323592	chr10:60108935	G>T	exon27:p.Arg1023Leu	exon28:p.Arg1017Leu	-	Stressman et al 2017
-	chr10:60105930	delC	exon28:p.Glu1102Serfs*16	exon29:p.Glu1096Serfs*16	de novo	Kloth et al 2021
-	chr10:60086763	C>T	exon30: p.Pro1221Leu	exon31:p.Pro1215Leu	inherited	Wang et al 2016
-	chr10:60086763	C>T	exon30:p.Pro1221Leu	exon31:p.Pro1215Leu	inherited	Stressman et al 2017
rs1589784865	chr10:60086698	C>T	exon30: p.Arg1243Cys	exon31 p.Arg1237Cys	inherited	Bonnet-Brilhault et al 2015
rs767721464	chr10:60086697	G>A	exon30:p.Arg1243His	exon31:p.Arg1237His	inherited	Stressman et al 2017
rs773581870	chr10:60083557	G>A	exon33:p.Gly1379R	exon34:p.Gly1373R	-	Stressman et al 2017
rs375050420	chr10:60076176	T>G	exon37: p.Ser1569Ala	intron36	de novo	Bi et al 2012
-	chr10:60074069	A>G	exon37: p.Met271Thr	intron36	de novo	Sanders et al 2012
-	chr10:60074069	T>C	exon37:p.Met2271Thr	intron36	de novo	Iossifov et al 2014
-	chr10:60073614	C>T	exon37: p.Arg2423Cys	intron36	de novo	Iossifov et al 2014
-	chr10:60073614	C>T	exon37:p.Arg2423Cys	intron36	de novo	Lim et al 2017
-	chr10:60069971	A>G	exon37: p.His3637Arg	intron36	inherited	codila sola et al 2015
rs879255535	chr10:60069886	delC	exon37:p.Thr3666fs*2	intron36	de novo	Iqbal et al 2013
-	chr10:60069813	G>A	exon37:p.Gly3690Arg	intron36	inherited	Shi et al 2013
-	chr10:60068722	C>T	exon37: p.Thr3720Met	intron36	inherited	Bi et al 2012
-	chr10:60055960	A>C	exon 37: p.Thr4255Pro	intron36	inherited	Bi et al 2012
